# Extremely High Fitting Range and Dangerous Levels Permitted by the Current FDA Guidelines for OTC Hearing Aids and the Need to Consider RECDs

**DOI:** 10.3390/audiolres16050124

**Published:** 2026-08-25

**Authors:** King Chung

**Affiliations:** 1 School of Clinical and Developmental Sciences, College of Health and Human Sciences, Northern Illinois University, Dekalb, IL 60115, USA; kchung@niu.edu; 2 Department of Communication Sciences and Disorders, School of Health and Rehabilitative Sciences, MGH Institute of Health Professions, Boston, MA 02129, USA; kchung1@mghihp.edu

**Keywords:** over-the-counter, OTC, hearing aids, FDA, guidelines, real-ear-to-coupler difference, RECD

## Abstract

**Background/Objective**: One of the missions of the United States FDA is “protecting the public health by ensuring the safety, efficacy, and security of human and veterinary drugs, biological products.” The current guidelines for OTC hearing aids impose an output limit of 111 dB SPL for linear hearing aids and 117 dB SPL for compression hearing aids when measured using 90 dB SPL pure tones in a 2 cc coupler. This means that the sound pressure level at the ear drum at a frequency would be 111 or 117 dB SPL plus the real-ear-to-coupler difference (RECD) of the 2 cc coupler at the frequency. **Methods**: The maximum aidable hearing thresholds by hypothetical OTC-compression hearing aids that are compliant with the current FDA guidelines were explored using NAL-NL2 and DSLv5 prescriptive methods. The theoretical maximum permissible overall output levels in the ear canal were also calculated. Additionally, the fitting range of OTC-compression hearing aids with a maximum of 117 dB SPL output level at the ear drum was explored. **Results**: The current FDA guidelines allow OTC-compression hearing aids to provide sufficient gain and headroom to fit individuals with severe to profound hearing loss. In the presence of a loud wideband signal, the maximum overall output level can reach 164.5 dB SPL in the ear canal for an OTC-compression hearing aid with a bandwidth of 5657 Hz. When the maximum output levels are defined at the ear drum (i.e., the RECD is already considered), however, the fitting range is lowered to moderate hearing loss and the maximum overall output level is lowered by 10 dB. **Conclusions**: These findings identified potential regulatory vulnerability in the current FDA guidelines and suggest the need to take the measurement coupler and its corresponding RECDs into account when setting the guidelines for OTC hearing aids. Other alternative strategies to prevent potential abuses of the guidelines are also discussed.

## 1. Introduction

The United States Food and Drug Administration (FDA) is responsible for developing guidelines to ensure public safety before the launch of any new medical devices and for reinforcing such guidelines in case of incompliance [1]. Globally, approximately 1.57 billion people have some degree of hearing loss [2] and ~90% of them have mild to moderate hearing loss [3]. In 2017, the FDA established over-the-counter (OTC) hearing aids as a new category of medical devices aiming to reduce barriers and improve access to amplification and to reduce the negative impact of untreated hearing loss [4]. Although the FDA’s definition for OTC hearing aid is to treat *perceived* mild to moderate hearing loss, Chung and Zeng [5] showed that the current guidelines could adequately fit individuals with 80 dB HL of flat sensorineural hearing loss. This paper further explores the maximum fitting range of OTC hearing aids, the maximum overall sound pressure level at the ear drum, and alternative considerations for the guidelines.

Hearing loss negatively impacts communication, physical and mental health, learning, income, and quality of life [6,7,8,9,10,11]. It is also associated with loneliness, depression, cognitive decline, high risk for cardiovascular disease, hypertension, hospitalizations, falls, and mortality among older adults [12,13,14,15,16,17,18]. Treating hearing loss, therefore, has become one of the global priorities for organizations such as the US National Academies of Sciences, Engineering, and Medicine [19], World Health Assembly [20], World Health Organization [21] and a Lancet Commission [22,23].

Amplification devices such as hearing aids and cochlear implants are effective options for managing the majority of chronic hearing loss cases. They improve the communication abilities of users, enable them to engage in more social interactions, and enhance their quality of life [24,25,26]. Recent studies also suggest that hearing loss is one of the two greatest modifiable causes for dementia later in life and mediating hearing loss in mid-life can reduce the world’s population with dementia by 7% [23]. Despite these benefits, individuals with hearing loss in the United States often wait 4 to more than 6 years to get their hearing aids after first noticing their hearing difficulties [27]. Individuals in other parts of the world may wait significantly longer before seeking help or they may never wear hearing aids—only 9–15% of individuals with hearing loss in low-to-middle income countries wear hearing aids [3]. Two of the major barriers often cited for not seeking amplification are: (1) the high cost of hearing aids because prescription hearing aids often cost thousands of dollars; and (2) difficulties accessing services because the hearing aid fitting process often takes several appointments with audiologists or hearing professionals [28,29,30,31]. The good news is that approximately 70% of users wished they had gotten their hearing aids sooner [32], indicating the positive impact of amplification in their lives.

In 2017, the FDA established a set of guidelines for OTC hearing aids to provide amplification for individuals with *perceived* mild to moderate hearing loss [4]. The American Speech-Language-Hearing Association defines a mild to moderate hearing loss as having hearing thresholds of 26–55 dB HL [33] and the World Health Organization as thresholds of 20–49 dB HL [3].

Before the final/current guidelines [34] went into effect, the FDA invited public comments. Experts from American Academy of Audiology, Academy of Doctors of Audiology, American Speech-Language-Hearing Association, and International Hearing Society formed a working group and jointly published the Consensus Paper from Hearing Care Associations [35]. Two of the five recommendations made to FDA were (1) to establish product requirements appropriate for OTC hearing aids targeting mild to moderate hearing impairment, and (2) to provide adequate consumer protection, in coordination with the Federal Trade Commission.

As part of the recommendations to establish production requirements appropriate for OTC hearing aids targeting mild to moderate hearing impairment, the four-organization working group recommended the FDA to require OTC hearing aid to have input compression with a volume control to reduce the likelihood of overamplification and loudness discomfort. Based on the gain targets of the hearing prescriptive methods developed by National Acoustics Laboratories (NAL-NL2 [36]), the working group also recommended to limit the average gain of OTC hearing aids at 1000, 1600 and 2500 Hz for pure tones presented at 50 dB SPL to be at or below 25 dB when the hearing aid is programmed to the maximum volume [37]. They also recommended limiting the coupling device to instant fit tips and requiring potential users to see an audiologist, should custom earmolds be needed.

In addition, the working group recommended limiting the maximum output level of OTC hearing aids with input compression to 110 dB SPL when measured using the ANSI/ASA S3.22:2014 (R2020) OSPL90 protocol. This is because the calculations of Johnson [38] revealed that, to avoid causing additional hearing loss due to overamplification, the outputs of hearing aids for individuals with moderate hearing loss should not exceed an overall level of 111 dB SPL in the ear canal for 8–8.5 h of daily usage. The OSPL90 testing protocol measures the maximum output levels of hearing aids in 2 cc couplers in response to pure tones presented at 90 dB SPL when the hearing aids are set to the maximum volume (i.e., full-on gain). As the OSPL90 is measured using pure tones presented one at a time from low to high frequency (i.e., a pure tone sweep), the working group also acknowledged that the overall level of the hearing aid in response to a complex signal would be much greater than 110 dB SPL.

Despite these recommendations, the FDA’s current guidelines do not limit the gain nor the type of coupling device. They allow a maximum output limit of 111 dB for OTC hearing aids with linear amplification (OTC-linear) and 117 dB for OTC hearing aids with input compression (OTC-compression) when measured in a 2 cc coupler [34]. One of the crucial aspects that is not mentioned in the FDA’s nor the joint working group’s documents, is the real-ear-to-coupler differences (RECDs). All the measurements in both documents were specified using the ANSI/ASA S3.22:2014 (R2020) standard [37], which uses the 2 cc coupler as the standard measurement coupler.

The 2 cc couplers have a volume of 2 cc. Yet the residue ear canal volume when a custom hearing aid is worn in the ear or when the output of a behind-the-ear hearing aid is coupled in the ear canal with an earmold or a dome, is typically between 0.5 and 1.5 cc, depending on the size of the ear canal and how deep the earmold/dome is placed in the ear canal [39]. The current FDA guidelines specify the insertion depth of the coupling device (e.g., dome or earmold) needs to be 10 mm or more from the ear drum, which leaves a volume of ~0.51 cc (calculated from Voss and colleagues [40], 0.616/12 × 10 = 0.51 cc). With RECDs considered, the maximum sound pressure level permitted at the ear drum is equal to the maximum level in a 2 cc coupler (i.e., 111 or 117 dB SPL) plus the RECDs of the 2 cc coupler at each frequency. After adding the RECD values, the sound pressure levels at the ear drum permitted by the current FDA guidelines become 120–140 dB SPL for OTC-compression hearing aids and 114–134 dB SPL for OTC-linear hearing aids [5].

Chung and Zeng [5] showed that OTC hearing aids that meet the current guidelines were able to fit individuals with 80 dB HL flat hearing loss monaurally. As NAL-NL2 prescribes 2 dB less gain at low input levels and up to 6 dB less gain at high input levels for binaural fittings compared to monaural fittings [36], OTC-compression hearing aids can potentially meet the prescriptive targets for binaural users with even higher degrees of hearing loss. These analysis findings raised additional questions about the limits of the current FDA guidelines: What is the maximum hearing loss that OTCs can fit? Are the guidelines suitable for individuals with mild to moderate hearing loss? With the goal to “regulate all hearing aids to ensure safety and effectiveness for consumers” [41], does the guidelines have potential vulnerability that would allow others to circumvent the existing safety regulations for prescription hearing aids to fit individuals with much higher degrees of hearing loss?

Currently, four of the big five hearing aid companies have marketed OTC hearing aids (e.g., ReSound’s Jabra) or jointly developed OTC hearing aids with another company (e.g., WS with Sony). They definitely have the capability and the know-how to design and market OTC hearing aids with the maximum output levels specified in the FDA current guidelines. Additionally, there are more than 200 amplification apps in the iOS App Store for APPLE smartphones and more than 30 apps in the Google Play Store for ANDROID smartphones. Many of them use earbuds to provide amplification. The frequency responses of traditional hearing aids are usually designed to have a prominent peak(s) between 2000 and 4000 Hz to compensate for the insertion loss [42]. The frequency responses of many earbuds, however, are relatively flat with amplitudes within 10 dB from low to high frequencies [43,44,45,46]. The effects of these relatively flat frequency responses on the overall levels of OTC hearing aids permitted by the current FDA guidelines and the maximum hearing loss they can fit have not been explored.

The purposes of the following analyses are to examine: (1) the maximum aidable hearing thresholds of OTC-compression hearing aids permitted by the current FDA hearing aid guidelines after applying the RECDs, (2) the maximum overall output sound pressure level in the 2 cc coupler and in the ear canal permissible by the current FDA guidelines, and (3) the maximum aidable hearing thresholds and the maximum overall output sound pressure level of an OTC-compression hearing aid if the 117 dB SPL limit is defined in the ear instead of in the 2 cc coupler (i.e., the effects of RECD is accounted for). OTC-compression hearing aids are chosen because most modern hearing aids have compression algorithms/circuits. The findings will shed light on the implications of the current FDA guidelines and provide evidence for alternative strategies to ensure the safety of OTC hearing aids.

## 2. Methods

### 2.1. Maximum Aidable Hearing Thresholds

The maximum aidable hearing thresholds were determined by using a VeriFit2 Hearing Aid Verification System (AudioScan, Dorchester, ON, Canada), which is capable of generating real ear aided response (REAR) targets at different input levels using several hearing aid fitting prescriptive methods based on the perspective user’s hearing thresholds [47]. The available hearing aid prescriptive methods include: NAL-NL2 [48], DSLv5 [49], CAM-REST [50], and CAMEQ2-HF [51]. Although both have children and adult versions, NAL-NL2 is the most used for fitting adults and DSLv5 for children [35]. As the FDA [34] indicated that they do not want to make decisions on the guidelines based on a particular fitting prescriptive method, both NAL-NL2 and DSLv5 are included in the following analyses.

The current FDA guidelines only specify the output limits of the OTC hearing aids and do not limit the maximum permissible gain. This means that the maximum sound pressure levels at the ear drum (i.e., in situ) across the audiometric frequencies are the limits for any hearing aid fitting. The maximum in situ sound pressure levels can be calculated by adding the RECD for 2 cc coupler at each frequency plus the maximum output permissible by the FDA guidelines (i.e., RECD + 111 dB for OTC-linear hearing aids, and RECD + 117 for OTC-compression hearing aids). Please see how RECDs are used in the hearing aid fitting process in Supplementary File S1.

In hearing aid fittings, the maximum in situ sound pressure levels are often determined by the uncomfortable loudness levels (UCLs) of the hearing aid user or the maximum power outputs (MPOs) of the hearing aids across frequencies. UCLs represent the sound pressure level at which the sound becomes uncomfortable to listen to and the MPOs represent the output levels that the hearing aid should not or cannot exceed at different frequencies. The MPO settings are usually tested using the OSPL90 protocol specified by ANSI/ASA S3.22-2014 (R2020) [37]. When the MPOs are not limited by the physical capabilities of the hearing aid, they are usually determined by the UCLs of the user and the term MPOs can be used interchangeably with UCLs in such cases. Both NAL-NL2 [48] and DSLv5 [49] recommend limiting the maximum output of the hearing aids based on the UCLs but NAL-NL2 uses the term UCLs and DSLv5 uses the term MPOs. To generate the fitting targets, the audiologist can enter the hearing aid user’s individual UCLs or apply average UCLs based on the user’s hearing thresholds.

Before the search for the maximum aidable hearing thresholds on VeriFit2, several other parameters also need to be considered. First, Verifit2 allows several different types of testing stimuli to be used in the fitting target generation process (e.g., pure tones, the “carrot passage”, or ISTS). The fitting targets generated by using different input stimuli are different because the stimuli have different spectral shapes. The International Speech Test Signal (ISTS) consists of words from six different languages spoken by six female talkers [52,53]. The ISTS was used as the speech stimuli in the current study because it has similar spectral shape as the International Long-Term Average Speech Spectrum.

Second, the hearing aid fitting targets can be generated for binaural or monaural fittings. The target gains are usually lower for binaural fittings because of loudness summation. However, the UCLs and MPOs estimated by both NAL-NL2 and DSLv5 are identical for monaural or binaural fittings. Monaural hearing aid fitting is used in the analyses with the understanding that the gains of binaural fittings are lower than those reported in this study. Other parameters used to generate the maximum aidable hearing thresholds include: (1) Insert + Foam as the transducer used to test hearing thresholds, (2) average RECD for adults, (3) non-tonal language for NAL-NL2, (4) BTE + custom mold as the hearing aid style, and (5) occluded earmold as the sound coupling device.

**Figure 1:**
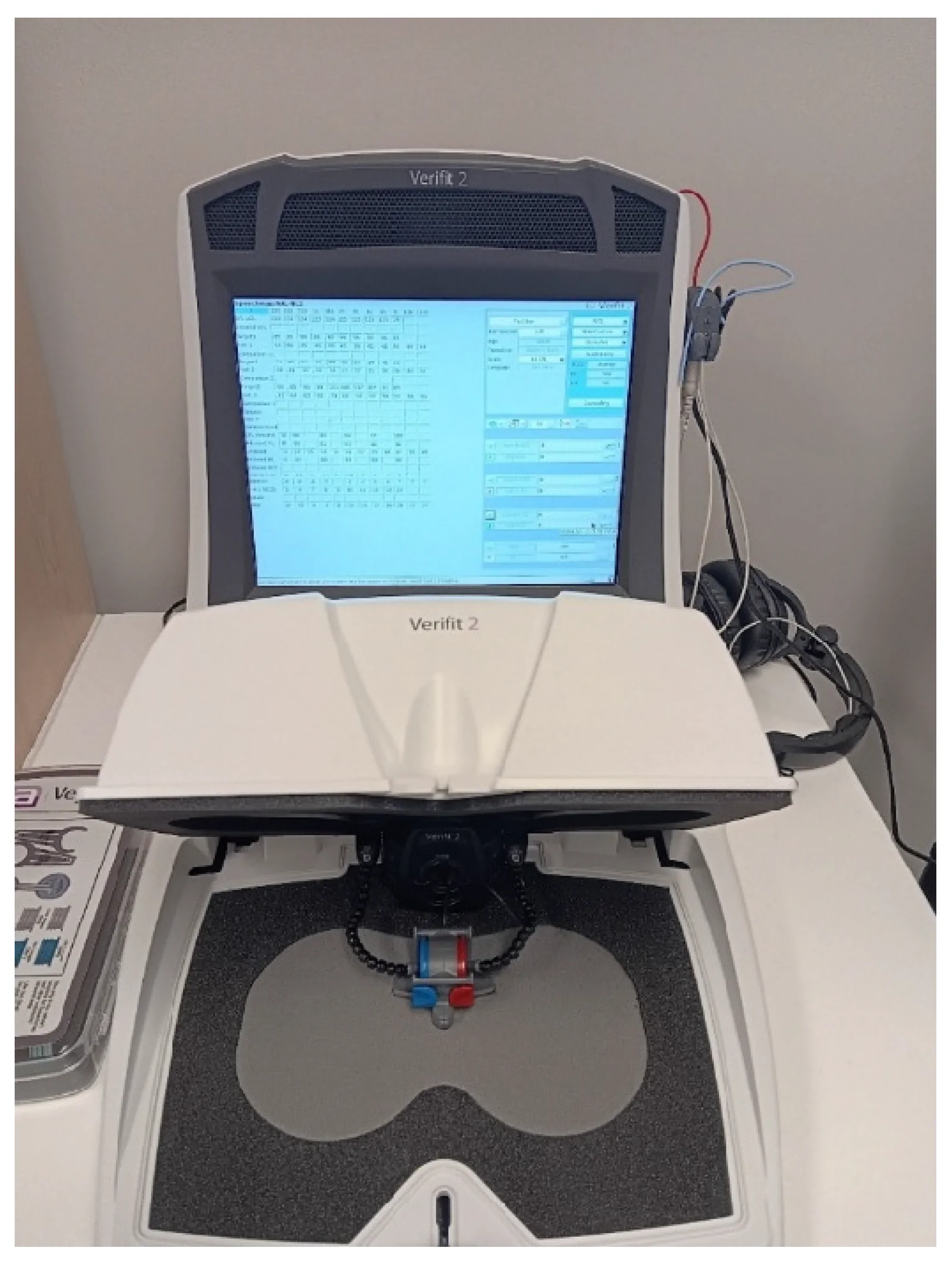
The setup for examining the prescriptive targets for different degrees of hearing loss using VeriFit 2.

To find the maximum aidable hearing threshold at each frequency, an 80 dB HL hearing threshold was entered into VeriFit2 (Figure 1) and the threshold was increased or decreased in 5 dB steps until the UCL predicted by each prescriptive method is just below the maximum sound pressure level permitted in the ear canal at the frequency.

After the maximum aidable thresholds were determined, the fitting targets for one-third octave bands at octave frequencies between 250 and 8000 Hz were obtained by presenting ISTS stimuli at 80, 65, and 50 dB SPL. The presentation level of the ISTS at each one-third octave band was measured by the microphones inside the center coupler module in the hearing aid test box (i.e., the microphones are located inside the gray part between the blue and red coupler covers in Figure 1). The reference microphones (i.e., the little arms pointing at the center coupler module) ensure that the loudspeakers are emitting the intended overall levels at the coupler entrances. The gain needed to achieve the maximum aidable hearing loss was calculated using the formula: Gain = Output − Input.

### 2.2. Maximum Overall Sound Pressure Levels Permitted in the 2 cc Coupler and the Ear Canal

In rare occasions, a loud noise can occur in the environment that can cause dangerous levels in the ear canal. An example is a white noise with an amplitude of 90 dB SPL level per cycle. As the output limits of OTC-compression hearing aids are defined and measured by presenting a 90 dB SPL pure tone sweep (i.e., presenting 90 dB SPL pure tones one at a time from low to high frequency), this means each component of the white noise is amplified to 117 dB SPL. The maximum permissible overall sound pressure levels by the current FDA guidelines are estimated using the equation for calculating the overall levels of wideband sounds:

Overall Level = Level per Cycle + 10log (BW)(1)

where

Level per Cycle = the sound pressure level at each frequency

BW = the bandwidth of the stimulus

Note that both ANSI/ASA S3.22-2014: R2020 [37] standard on specifications of hearing aid characteristics and ANSI/CTA standard [54] for personal sound amplification performance criteria specify the hearing aid frequency response up to 5000 Hz, above which the amplitude is not regulated. This means that the output levels can be higher or lower than the 111 and 117 limits at frequencies above 5000 Hz. As many hearing aids have a bandwidth between 5000 and 6000 Hz and the earbuds are capable of providing relatively flat frequency response to at least 8000 Hz and, in some cases, to >10,000 Hz [43,44,45,46,55,56], the maximum overall sound pressure levels are calculated for hearing aid bandwidths of 111–5756 Hz and 111–8980 Hz. Please see Supplementary File S2 and Supplementary Table S1 for detailed procedures.

According to National Institute for Occupational Safety and Health [57], every 3 dB of increase in level doubles the acoustic sound energy hitting the ear and the exposure time needs to be halved. Johnson [38] reported an 8 h safe levels of 111 dB SPL for individuals with 55 dB HL of moderate hearing loss. The duration at which an individual with moderate degree of hearing loss can safely listen at the maximum overall output level without incurring permanent hearing loss was calculated using a 3 dB trade-off rule for hearing aids with a bandwidth of 111–5657 Hz. Please see the detailed step-by-step calculations in Supplementary File S3.

### 2.3. What if the 117 dB SPL Output Limit Is Defined After Taking RECD into Account?

One of the alternatives and simple ways of revising the guidelines is to incorporate the RECDs in the 117 dB SPL output limit of the OTC-compression hearing aids. In other words, the maximum output level of the OTC-compression measured in the ear canal is 117 dB at each frequency, and the maximum output level measured in a coupler equals: 117 minus RECD at each frequency. The maximum aidable hearing thresholds and the maximum overall levels were determined using similar procedures as described in Section 2.1 and Section 2.2. The exposure time was also calculated using the 3 dB trade-off rule.

## 3. Results

### 3.1. Maximum Aidable Hearing Thresholds

The maximum sound pressure level permitted at the ear drum across the audiometric frequencies varied from 120 to 140 dB SPL (Row C of Table 1). With the RECDs of the 2 cc couplers taken into account, the maximum aidable hearing thresholds range from 75 to 110 dB HL according to NAL-NL2 (Row D of Table 1). The four-frequency pure tone average (i.e., PTA4 = average thresholds at 500, 1000, 2000, and 4000 Hz) equals 82.5 dB HL. These thresholds and the PTA4 are 20–55 dB higher than individuals with a maximum moderate hearing loss of 55 dB HL. The permissible amount of gain varied from 37 to 60 dB for ISTS stimuli presented at 50 dB SPL input (Row O), 32 to 59 dB at 65 dB SPL (Row L), and 26 to 58 dB at 80 dB SPL (Row I), which are in the power hearing aid range.

Note that, normally, the one-third octave band sound pressure levels increase for 15 dB at all frequencies when the overall input level of the stimuli increases by 15 dB (i.e., from Row L to Row I for 50–65 dB overall levels, respectively). However, when the overall level increased from 65 to 80 dB, the one-third octave band levels increased between 8 and 22 dB (i.e., Row I–Row H). This is because VeriFit2 automatically applies a high frequency emphasis to speech with overall levels higher than 75 dB SPL to mimic the spectrum of loud speech [58].

The headroom prescribed by NAL-NL2 was calculated by subtracting the REAR targets for 80 dB inputs (Row G in Table 1) from the estimated UCLs in dB SPL (Row E). The headroom estimated by NAL-NL2 was 13–42 dB above the REAR targets of 80 dB input, which is more than adequate considering that the overall levels of speech peaks are only ~12 dB higher than the overall level of the long-term average speech spectrum (LTASS, i.e., crest factor = 12 dB) [57] and the crest factor for music is +18 dB [39].

Table 2 depicts similar results when DSLv5 was used as the hearing aid prescriptive method. The values on Row D indicate that the current FDA guidelines allow OTC-compression hearing aids to provide adequate amplification for adult users with hearing thresholds in the range of 85–125 dB HL with a PTA4 of 95 dB HL. These findings are even higher than those generated by NAL-NL2. The amount of gain required for individuals with such degree of hearing loss ranged from 47 to 73 dB for inputs at 50 dB SPL, 47–77 dB at 65 dB SPL, and 43–68 dB at 80 dB SPL. Notice that the headroom across frequencies that DSLv5 reserves are between 7 and 15 dB, which are less than those for NAL-NL2.

### 3.2. Maximum Permissible Overall Sound Pressure Levels in the 2 cc Coupler and in the Ear Canal

Table 3 Column G shows the maximum hearing aid overall output level permissible by the current FDA guidelines for an OTC-compression hearing aid with flat frequency response and different bandwidths when measured in a 2 cc coupler (e.g., from 111 to 5657 Hz is 154.4 dB SPL and from 111 to 8980 Hz is 156.6 dB SPL). Table 3 Column J shows the overall levels in an average ear by adding the RECD. The overall levels for these bandwidths become 164.6 dB SPL and 170.0 dB SPL, respectively.

Using the 3 dB trade-off rule, the safe listening time for a hearing aid with bandwidth of 111–5657 Hz (i.e., 164.6 dB SPL) would be 0.2 s.

### 3.3. What if the 117 dB SPL Output Limit Is Defined After Taking RECD into Account?

Row D in Table 4 and Table 5 show the maximum aidable hearing thresholds for hearing aids with a maximum output level of 117 dB SPL in the ear canal using NAL-NL2 and DSLv5 prescriptive methods, respectively. The fitting range generated using NAL-NL2 is 75 dB HL at 250 and 50–60 dB at higher frequencies (Table 4 Row D). The PTA4 is 62.5 dB HL which is only 7.5 dB higher than the maximum moderate hearing loss of 55 dB HL. When the DSLv5 fitting prescription is used, the maximum aidable hearing loss is 75 dB HL at 250 Hz, 50–60 dB HL in mid-frequencies, and 85 dB HL at 8000 Hz (Table 5 Row D). The PTA4 equals 55 dB HL, which is the maximum moderate hearing loss.

The headroom of the hearing aid fitting is calculated by subtracting Row G from Row E and tabulated in Row F (Table 4 for NAL-NL2; and Table 5 for DSLv5). The resulting headrooms for both prescriptive methods are sufficient for listening to loud speech with an r.m.s. of 80 dB SPL without distortion. The headrooms can also accommodate music with an r.m.s. of 80 dB SPL without distortion. As NAL-NL2 generally calls for higher compression ratios than DSLv5, the headrooms for NAL-NL2 are generally higher than those of DSLv5 across frequencies.

If 117 dB SPL is the maximum hearing aid output level permitted in the ear canal instead of in 2 cc coupler, the following labels should replace the first row of labels in Table 3:

- Column E: Maximum Hearing Aid Output Level Permitted at Each Frequency in the Ear (dB SPL)
- Column F: Total Band Level in the Ear (dB SPL)
- Column G: Overall Output Level for BW from 111 to f_H_ Hz in the Ear (dB SPL)

The maximum overall level is 154.4 dB for an OTC-compression hearing aid with 5657 Hz bandwidth. This level is still very high, but it is 10 dB less than the maximum overall level permitted by the current FDA guidelines (i.e., Table 3 Column J). Calculated using the 3 dB trade-off rule, the listening time was increased to 1.3 s.

## 4. Discussion

Research studies conducted to examine commercially available OTC hearing aids typically recruit participants with mild to moderate hearing loss and they generally reported positive results with some areas for improvement [59,60,61,62,63]. Specifically, Sabin and colleagues [59] reported that their participants were able to self-program their OTC hearing aids and, after a month-long trial, the self-fit group obtained higher sound quality ratings than the group fit by experienced audiologists and there was no difference in speech in noise performance between the two groups. Knoetze and colleagues [60] conducted a study to compare the gain settings of six self-fit OTC hearing aids with the NAL-NL2 targets. They found that self-adjusted gains of the OTC hearing aids typically were set slightly lower than the target gains, especially in higher frequencies. When the efficacy was compared between self-fit and audiologist-fit OTC hearing aids of the same model, participants in the self-fit group reported higher subjective ratings at two weeks but no significant differences in subjective ratings at six weeks [61]. No significant difference was reported in the objective digit-in-noise test scores during the course of the trial. In the MarkeTrack report, Picou and Huang [62] reported that patient satisfaction was similar between prescriptive and OTC hearing aids. These findings indicate that at least some of the currently commercially available OTC hearing aids can be viable lower cost alternatives to prescriptive hearing aids for individuals with mild to moderate hearing loss.

In this study, the maximum aidable hearing thresholds of hypothetical OTC-compression hearing aids that are built to have the maximum levels permitted by the current FDA guidelines were examined without gain restrictions. Row D of Table 1 and Table 2 showed that the current FDA guidelines allow OTC-compression hearing aids to provide sufficient gain to fit individuals with severe to profound hearing loss as long as they claim to have *perceived* mild to moderate hearing difficulties and have custom earmolds. In addition, the maximum overall output sound pressure levels permitted at the ear drum can reach 164 dB for OTC-compression hearing aids with a bandwidth of 111 to 5657 Hz and 170 dB SPL for those with a bandwidth of 111 to 8980 Hz (Table 3 Column J). These levels are equivalent to firing a 12-gauge shotgun without wearing hearing protection [64] and they are dangerously high for any human ears to be exposed to. Even a brief moment of exposure would likely incur structural damage to the middle and inner ear, tinnitus, and/or permanent hearing loss [65,66,67,68]. By using the 3 dB trade-off rule, the exposure level for a hearing aid with 111–5657 Hz is only 0.2 s, which does not allow time for the user to remove the hearing aid or reduce the gain before inducing permanent hearing loss. Fortunately, such loud noise rarely happens in daily lives but the level is dangerously high if it does.

One may argue that current OTC hearing aids are relatively safe [69] and no one would create OTC hearing aids with the maximum levels permitted by the current FDA guidelines. Yet, creating such hearing aids is not too far-fetched because four of the world’s largest hearing aid manufacturers also market OTC hearing aids and studies have reported that the OSPL90 of personal sound amplification products (PSAPs) were mostly between 110 and 120 dB SPL and some could exceed 130 dB SPL [70,71]. Additionally, some earbuds have relatively flat frequency responses extending beyond 10,000 Hz. These results expose a regulatory vulnerability in the current FDA guidelines to permit others to circumvent the lengthier regulatory process for prescription hearing aids (Class II medical devices) and to market OTC hearing aids to individuals with severe to profound hearing loss.

Although allowing OTC hearing aids the capability to provide amplification to individuals with severe to profound hearing loss can help more people in need, their use might also unintentionally create missed the opportunities for better hearing care because individuals with higher degree of hearing loss are more likely to (1) experience difficulties hearing and understanding speech in background noise [72,73], and (2) have more psychophysically measured loss in addition to the loss of hearing sensitivity (e.g., longer gap detection and worse frequency selectivity) [74,75]. They may benefit from using more advanced noise reduction technologies and algorithms that are readily available in prescription hearing aids, e.g., adaptive directional microphones, remote microphones, and artificial-intelligence-based noise reduction algorithms.

One of the possible ways to retain the mission of “fostering innovation” (i.e., do not limit gain) but to bring the fitting range to mild to moderate hearing loss range is to account for RECDs in the guidelines. When the 117 dB SPL is used as the maximum output level in the ear canal (i.e., UCLs, Row C Table 4 and Table 5), OTC-compression hearing aids can provide sufficient amplification and ample headroom for speech and music for individuals with moderate (i.e., PTA4 of 55 dB HL by DSLv5, Row D in Table 5) or slightly higher than moderate hearing loss (i.e., PTA4 of 62.5 dB HL by NAL-NL, Row D in Table 4). The slightly higher aidable hearing thresholds at 250 Hz and 8000 Hz would still permit individuals with *perceived* mild to moderate hearing loss some leeway and flexibility to use OTC hearing aids.

Moving forward, the FDA can take one or multiple of the following parameters into consideration and revise their current OTC hearing aid guidelines to reduce the regulatory vulnerability and to increase patient safety:

1. To impose a realistic gain limit for the intended populations across frequencies,
2. To define the maximum output level in the ear canal and derive the maximum output levels in 2 cc or 0.4 cc coupler after considering their RECDs. The new IEC 60318-8:2022 [76] standard has incorporated the 0.4 cc coupler which is the default coupler used in VeriFit2, and
3. To restrict the coupling devices to be instant fit options (e.g., domes), which serve as a secondary measure to limit the gain of the OTC hearing aids. This is one of the recommendations of the four-organization working group [35]. The rationale is that these instant-fit coupling devices do not allow high amount of gain before feedback, making OTC hearing aids feasible for individuals with mild to moderate hearing loss but not for individuals with severe to profound hearing loss.

As the availability of OTC hearing aids is likely to increase in the US and in other countries, regulatory agencies in other countries may rely on the existing guidelines to inform the development of their own guidelines. The extremely high fitting range and the dangerous overall levels permitted by the current FDA guidelines need to be known and addressed so that OTC hearing aids can be effectively regulated to serve as safe alternatives to prescription hearing aids for individuals with mild to moderate hearing loss around the world.

## Figures and Tables

**Table 1:** The maximum aidable hearing thresholds permitted by the current FDA guidelines using the NAL-NL2 prescriptive method.

Row	Frequency (Hz)	250	500	1000	2000	4000	8000
**A**	**Maximum Hearing Aid Output Level Permitted in 2 cc Coupler** **(dB SPL)**	**117**	**117**	**117**	**117**	**117**	**117**
**B**	**RECD of 2 cc Coupler (dB)**	3	4	9	10	15	23
**C = A + B**	**Maximum Hearing Aid Output Level Permitted in the Ear** **(dB SPL)**	120	121	126	127	132	140
**D**	**Maximum Aidable Hearing Threshold (dB HL)**	**80**	**75**	**85**	**80**	**90**	**110**
**E**	**In-Situ UCL Prescribed by** **NAL-NL2 (dB SPL)**	118	121	126	125	130	140
**F = E − G**	**Headroom (dB)**	30	27	29	13	12	42
**Overall ISTS at 80 dB Input**							
**G**	**Target 80 (dB SPL)**	88	94	97	112	118	98
**H**	**Input Level at 1/3 Octave Band** **(dB SPL)**	62	64	65	70	60	58
**I = G − H**	**Gain 80 (dB)**	26	30	32	42	58	40
**Overall ISTS at 65 dB Input**							
**J**	**Target 65 (dB SPL)**	86	89	87	100	101	92
**K**	**Input Level at 1/3 Octave Band** **(dB SPL)**	54	50	44	48	42	50
**L = J − K**	**Gain 65 (dB)**	32	39	43	52	59	42
**Overall ISTS at 50 dB Input**							
**M**	**Target 50 (dB SPL)**	76	81	80	92	91	78
**N**	**Input Level at 1/3 Octave Band** **(dB SPL)**	39	35	30	33	31	40
**O = M − N**	**Gain 50 (dB)**	37	46	50	59	60	38

**Table 2:** The maximum aidable hearing thresholds permitted by the current FDA guidelines using the DSLv5 prescriptive method.

Row	Frequency (Hz)	250	500	1000	2000	4000	8000
**A**	**Maximum Hearing Aid Output Level Permitted in 2 cc** **Coupler (dB SPL)**	**117**	**117**	**117**	**117**	**117**	**117**
**B**	**RECD of 2 cc Coupler (dB)**	3	4	9	10	15	23
**C = A + B**	**Maximum Hearing Aid Output Level Permitted in the Ear** **(dB SPL)**	120	121	126	127	132	140
**D**	**Maximum Aidable Hearing Threshold (dB HL)**	**85**	**85**	**100**	**90**	**105**	**125**
**E**	**In-Situ UCL Prescribed by** **DSLv5 (dB SPL)**	120	119	125	126	132	135
**F = C − D**	**Headroom (dB)**	15	9	10	7	7	9
**Overall ISTS at 80 dB Input**							
**G**	**Target 80 (dB SPL)**	105	110	115	119	125	126
**H**	**Input Level at 1/3 Octave Band (dB SPL)**	62	64	65	70	60	58
**I = G − H**	**Gain 80 (dB)**	43	46	50	49	65	68
**Overall ISTS at 65 dB Input**							
**J**	**Target 65 (dB SPL)**	101	103	106	109	119	122
**K**	**Input Level at 1/3 Octave Band (dB SPL)**	54	50	44	48	42	50
**L = J − K**	**Gain 65 (dB)**	47	53	62	61	77	72
**Overall ISTS at 50 dB Input**							
**M**	**Target 50 (dB SPL)**	86	88	91	94	104	107
**N**	**Input Level at 1/3 Octave Band (dB SPL)**	39	35	30	33	31	40
**O = M − N**	**Gain 50 (dB)**	47	53	61	61	73	67

**Table 3:** Maximum Hearing Aid Output Level in 2 cc Coupler and in the Ear.

Lower Cutoff Freq, f_L_ (Hz)	Center Freq of 1/3 OB, f_C_ (Hz)	Higher Cutoff Freq, f_H_ (Hz)	10 × log(BW) (dB SPL)	Maximum Output Level Permitted at Each Freq in 2 cc Coupler (dB SPL)	Total Band Level in 2 cc Coupler (dB SPL)	Overall Output Level for BW from 111 to f_H_ Hz in 2 cc Coupler (dB SPL)	RECD of 2 cc Coupler (dB)	Total Level in 2 cc Coupler + RECD (dB SPL)	Overall Output Level for BW from 111 to f_H_ Hz in the Ear (dB SPL)
A	B	C	D	E	F = E + D	G	H	I = F + H	J
111	125	140	14.6	117	131.6	131.6	3.5	135.1	135.1
140	160	177	15.7	117	132.7	135.2	3.5	136.2	138.7
177	200	222	16.5	117	133.5	137.5	3.5	137.0	141.0
222	250	281	17.7	117	134.7	139.3	3.5	138.2	142.8
281	315	354	18.6	117	135.6	140.9	3.5	139.1	144.4
354	400	445	19.6	117	136.6	142.2	3.5	140.1	145.7
445	500	561	20.6	117	137.6	143.5	3.6	141.2	147.1
561	630	707	21.6	117	138.6	144.8	4	142.6	148.4
707	800	890	22.6	117	139.6	145.9	4.5	144.1	149.8
890	1000	1122	23.7	117	140.7	147.0	5	145.7	151.2
1122	1250	1414	24.7	117	141.7	148.1	5.7	147.4	152.7
1414	1600	1782	25.7	117	142.7	149.2	6.3	149.0	154.2
1782	2000	2245	26.7	117	143.7	150.3	7.1	150.8	155.8
2245	2500	2828	27.7	117	144.7	151.3	8.5	153.2	157.7
2828	3150	3564	28.7	117	145.7	152.4	10	155.7	159.8
3564	4000	4490	29.7	117	146.7	153.4	11.5	158.2	162.1
4490	5000	5657	30.7	117	147.7	154.4	13.4	161.1	164.6
5657	6300	7127	31.7	117	148.7	155.5	15.2	163.9	167.3
7127	8000	8980	32.7	117	149.7	156.5	17	166.7	170.0
When the 117 dB SPL is defined as the hearing aid output limit in the ear canal, the labels of Column E, F, and G become:				**Maximum Output Level** **Permitted at Each** **Freq in the Ear** **(dB SPL)**	**Total Band Level in Ear (dB SPL)**	**Overall Output Level for BW from 111 to f_H_ Hz in the Ear (dB SPL)**			

**Table 4:** The maximum aidable hearing thresholds obtained by using the NAL-NL2 prescriptive method and treating 117 dB SPL as the maximum hearing aid output level permitted in the ear.

Row	Frequency (Hz)	250	500	1000	2000	4000	8000
**A**	**Maximum Hearing Aid Output Level Permitted in 2 cc Coupler** **(dB SPL)**	**114**	**113**	**108**	**107**	**102**	**94**
**B**	**RECD of 2 cc Coupler (dB)**	3	4	9	10	15	23
**C = A + B**	**Maximum Hearing Aid Output Level Permitted in the Ear** **(dB SPL)**	117	117	117	117	117	117
**D**	**Maximum Aidable Hearing Threshold (dB HL)**	**75**	**65**	**65**	**60**	**60**	**60**
**E**	**In-Situ UCL Prescribed by** **NAL-NL2 (dB SPL)**	115	114	116	115	115	115
**F = E − G**	**Headroom (dB)**	41	34	32	23	22	41
**Overall ISTS at 80 dB Input**							
**G**	**Target 80 (dB SPL)**	74	80	84	92	93	74
**H**	**Input Level at 1/3 Octave Band (dB SPL)**	62	64	65	70	60	58
**I = G − H**	**Gain 80 (dB)**	12	16	19	22	33	16
**Overall ISTS at 65 dB Input**							
**J**	**Target 65 (dB SPL)**	75	78	75	83	81	72
**K**	**Input Level at 1/3 Octave Band (dB SPL)**	54	50	44	48	42	50
**L = J − K**	**Gain 65 (dB)**	21	28	31	35	39	22
**Overall ISTS at 50 dB Input**							
**M**	**Target 50 (dB SPL)**	65	71	69	76	72	62
**N**	**Input Level at 1/3 Octave Band (dB SPL)**	39	35	30	33	31	40
**O = M − N**	**Gain 50 (dB)**	26	36	39	43	41	21

**Table 5:** The maximum aidable hearing thresholds obtained by using the DSLv5 prescriptive method and treating 117 dB SPL as the maximum hearing aid output level permitted in the ear.

Row	Frequency (Hz)	250	500	1000	2000	4000	8000
**A**	**Maximum Hearing Aid Output Level Permitted in the 2 cc** **Coupler (dB SPL)**	**114**	**113**	**108**	**107**	**102**	**94**
**B**	**RECD of 2 cc Coupler (dB)**	3	4	9	10	15	23
**C = A + B**	**Maximum Hearing Aid Output Level Permitted in the Ear** **(dB SPL)**	117	117	117	117	117	117
**D**	**Maximum Aidable Hearing Threshold (dB HL)**	**75**	**55**	**60**	**50**	**60**	**85**
**E**	**In-Situ UCL Prescribed by DSLv5 (dB SPL)**	116	116	115	116	116	115
**F = E − G**	**Headroom (dB)**	22	24	24	21	19	25
**Overall ISTS at 80 dB Input**							
**G**	**Target 80 (dB SPL)**	94	92	91	95	97	90
**H**	**Input Level at 1/3 Octave Band** **(dB SPL)**	62	64	65	70	60	58
**I = G − H**	**Gain 80 (dB)**	32	28	36	25	37	32
**Overall ISTS at 65 dB Input**							
**J**	**Target 65 (dB SPL)**	92	85	79	79	85	83
**K**	**Input Level at 1/3 Octave Band** **(dB SPL)**	54	50	44	48	42	50
**L = J − K**	**Gain 65 (dB)**	38	35	35	31	43	33
**Overall ISTS at 50 dB Input**							
**M**	**Target 50 (dB SPL)**	77	72	67	67	72	71
**N**	**Input Level at 1/3 Octave Band** **(dB SPL)**	39	35	30	33	31	40
**O = M − N**	**Gain 50 (dB)**	38	37	37	34	41	31

## Data Availability

The original contributions presented in this study are included in the article and Supplementary Materials. Further inquiries can be directed to the corresponding author. The author is willing to deposit Supplementary Table S1, the Excel file with calculations conducted in this study, into a public depository.

## Supplementary Materials

The following supporting information can be downloaded at: https://www.mdpi.com/article/10.3390/audiolres16050124/s1, File S1: Use of RECD in the Hearing Aid Fitting Process; File S2: Step-by-Step Calculations of the Permissible Maximum Overall Output Levels of OTC-Compression Hearing Aids Permitted by the Current FDA Guidelines; File S3: Calculations of the Usage Time for a User with Moderate Hearing Loss; Table S1: Calculations of Max Overall Output Levels of OTC-Compresshion Hearing Aids with Different Bandwidths.
