# Supplementary material for: Extremely High Fitting Range and Dangerous Levels Permitted by the Current FDA Guidelines for OTC Hearing Aids and the Need to Consider RECDs
Source: Audiol Res. 2026 Aug 25;16(5):124. doi: 10.3390/audiolres16050124 (PMC13600033; doi:10.3390/audiolres16050124)
Supplement: Supplementary file 1 [file audiolres-16-00124-s001.zip › audiolres-4292402-Supplementary Files S1-S3.pdf]

Supplementary File S1

Uses of RECD in the Hearing Aid Fitting Process

Audiometers are calibrated to present pure tones and speech levels in dB HL. In a hearing aid fitting, a hearing aid analyzer or a real-ear measurement equipment such as VeriFit2 uses the real-ear-to-coupler difference (RECD) in three conversions to generate the recommended level in dB SPL at each audiometric frequency [47]:

1) Take the hearing thresholds measured using different transducers, apply appropriate reference equivalent threshold sound pressure level (RETSPL) of the transducer, and add the RECD to convert the hearing threshold in dB HL to dB SPL, i.e.,

   Threshold in dB SPL = Threshold in dB HL + RETSPL + RECD … (a)

2) Add the RECD to the gain/output recommended by the fitting prescription and the microphone location effect (MLE) to generate the fitting targets (i.e., real ear aided response, REAR) for the speech stimuli at different input levels, e.g.,

   REAR for 50 dB input = Prescribed Output with 50 dB input + MLE + RECD … (b)

   where REAR is the real ear aided response that shows the frequency response of an amplification device when worn and measured in the ear canal

3) Take the uncomfortable loudness levels (UCLs) of the user or the average UCLs from a population, apply the appropriate RETSPL, and add RECD to generate the maximum power output (MPO) for the hearing aid (Mueller et al., 2021), i.e.,

   MPO in dB SPL = UCL in dB HL + RETSPL + RECD ………………. (c)

In VeriFit 2, UCLs after the conversion in Formula 3 are displayed (i.e., the MPO is displayed as UCLs in dB SPL).

Supplementary File S2

Step-by-Step Calculations of the Permissible Maximum Overall Output Levels of OTC-Compression Hearing Aids Permitted by the Current FDA Guidelines

Several steps were taken to calculate the maximum overall levels measured in a 2 cc coupler and in an average ear canal:

1. The cutoff frequencies of each one-third octave band with center frequencies (f_C) from 125-8000 Hz are calculate by:

   Low cutoff frequency(f_L) = f_C x 2^(-1/6) ………………..(d)

   High cutoff frequency (f_H) = f_C x 2^(1/6) ………………..(e)

   The high cutoff frequency of a lower center frequency (e.g., 125 Hz) also becomes the low cutoff frequency of the next center frequency (e.g., 160 Hz). So only the high cutoff frequency for the subsequent frequency bands are needed.

2. Overall Level = Level per Cycle + 10log (BW). The quantity of 10log (BW) is equal to 10log(f_H-f_L), which was calculated for each one-third octave band from 125 to 8000 Hz (Table 3 Columns B);

3. The total sound pressure level of each one-third octave band is calculated using the Formula Overall Level = Level per Cycle + 10log (BW) is equivalent to Column F = Column E + Column D in Table 3:

   Total Level in Each One-Third Octave Band = 117 + 10log(BW);

   where 117 = Maximum Level Permitted by FDA at Each Frequency

4. The overall level of the OTC-compression hearing aid with a bandwidth from 111 Hz to the f_H of the desired one-third octave band is calculated as:

   $I_z = 10\log(10^{Ix/10} + 10^{Iy/10})$ ………………………………….……… (f)

   where Ix is the intensity of the Signal X

Iy is the intensity of the Signal Y

Iz is the overall intensity of Signal X and Signal Y

and tabulated in Table 3 Column G.

Note that in the lowest one-third octave at the center frequency of 125 Hz, its bandwidth is 111-140 Hz, the overall level from 111-140 Hz (Column G) is equal to the total level in the band (Column F).

For all the other one-third octave bands, Iz in Formula f is the overall output level of the OTC hearing aid for the bandwidth from 111 to f_H Hz. Ix is the overall level in the previous bandwidth and Iy the total level in the additional bandwidth. For example, to calculate the overall level of an OTC with a bandwidth of 111-5667 Hz (i.e., f_C=5000 Hz),

* $Iz = 10\log(10^{153.4/10} + 10^{147.7/10}) = 154.4$ dB SPL

Note*: the values are obtained from the cells highlighted in purple in Table 3

Alternatively, the overall level can be calculated using the summation of the intensity at all the one-third octave bands:

$$Iz = 10\log\left(\sum_{i=125}^{fc} 10^{Ii/10}\right) \quad \ldots\ldots\ldots\ldots\ldots\ldots\ldots\ldots\ldots\ldots\ldots\ldots(g)$$

where Ii is the total levels of the one-third octave bands below and at the desired bandwidth

f_C is the highest center frequency of the desired bandwidth

Iz is the overall intensity of all the one-third octave bands within the desired bandwidth

For example, the overall level of an OTC hearing aid with a bandwidth of 111 to 281 Hz is the summation of the intensities in the one-third octave bands with center frequencies of 125-250 Hz (highlighted in purple in Table 3 Column I):

$$Iz = 10\log\left(\sum_{i=125}^{250} 10^{Ii/10}\right)$$

* $$Iz = 10\log(10^{131.6/10} + 10^{132.7/10} + 10^{133.5/10} + 10^{134.7/10})$$

$$Iz = 139.3 \text{ dB SPL}$$

Note*: the values are obtained from the cells highlighted in purple in Table 3

5. To calculate the total sound pressure levels with different bandwidths in the ear canal, the RECD values were added to Column F (i.e., Table 3 Column I = Column F + Column H); and then
6. The overall output levels for OTC-compression hearing aids with different bandwidths were calculated using Formula f or Formula g in a similar manner as delineated in Step 4. Their values are tabulated in Column J.

Supplementary File S3

Calculations of the Usage Time for a User with Moderate Hearing Loss

According to Table 3 of this current study, the maximum overall level permitted at the ear drum for a hearing aid with a bandwidth of 111-5667 Hz is 164.6 with RECD taken into account. According to Table 1 of Johnson [38], the safe level for an individual with 55 dB HL of hearing loss is 111 dB for 8 hours of usage before inducing 3.5 dB of permanent hearing loss. The usage time for a signal at 164.6 dB SPL (round up to 165 dB SPL) is calculated using a 3 dB between a sound pressure level and exposure time trade-off in several steps:

1. The number of dB exceeding the safe level is:

   165 – 111 = 54 dB

2. The number of times that the 8-hour time limit needs to be halved is equal to:

   54/3 = 18 times

3. The 8 hours is converted to seconds:

   8 x 3600 = 28888 seconds

4. The permissible time in seconds is:

   8 hrs x 3600 / (2^{18}) = 0.11 seconds

The usage time for a signal at 154.4 dB SPL is calculated without rounding using a 3 dB trade-off in several steps:

1. The number of dB exceeding the safe level is:

154.4 – 111 = 43 dB

2. The number of times that the 8-hour time limit needs to be halved is equal to:

   $43.4/3 \approx 14.46$ times

3. The 8 hours is converted to seconds:

   $8 \times 3600 = 28888$ seconds

4. The permissible time in seconds is:

   8 hrs $\times$ 3600 / $(2^{14.46})$ = 1.3 seconds
